# The Prognostic Value of the Work Ability Index for Sickness Absence among Office Workers

**DOI:** 10.1371/journal.pone.0126969

**Published:** 2015-05-27

**Authors:** Kerstin G. Reeuwijk, Suzan J. W. Robroek, Maurice A. J. Niessen, Roderik A. Kraaijenhagen, Yvonne Vergouwe, Alex Burdorf

**Affiliations:** 1 Department of Public Health, Erasmus MC, Rotterdam, The Netherlands; 2 NIPED Research Foundation, Amsterdam, The Netherlands; University of Louisville, UNITED STATES

## Abstract

**Background:**

The work ability index (WAI) is a frequently used tool in occupational health to identify workers at risk for a reduced work performance and for work-related disability. However, information about the prognostic value of the WAI to identify workers at risk for sickness absence is scarce.

**Objectives:**

To investigate the prognostic value of the WAI for sickness absence, and whether the discriminative ability differs across demographic subgroups.

**Methods:**

At baseline, the WAI (score 7-49) was assessed among 1,331 office workers from a Dutch financial service company. Sickness absence was registered during 12-months follow-up and categorised as 0 days, 0<days<5, 5≤days<15, and ≥15 days in one year. Associations between WAI and sickness absence were estimated by multinomial regression analyses. Discriminative ability of the WAI was assessed by the Area Under the Curve (AUC) and Ordinal c-index (ORC). Test characteristics were determined for dichotomised outcomes. Additional analyses were performed for separate WAI dimensions, and subgroup analyses for demographic groups.

**Results:**

A lower WAI was associated with sickness absence (≥15 days vs. 0 days: per point lower WAI score OR=1.27; 95%CI 1.21-1.33). The WAI showed reasonable ability to discriminate between categories of sickness absence (ORC=0.65; 95%CI 0.63-0.68). Highest discrimination was found for comparing workers with ≥15 sick days with 0 sick days (AUC=0.77) or with 1-5 sick days (AUC=0.69). At the cut-off for poor work ability (WAI≤27) the sensitivity to identify workers at risk for ≥15 sick days was 7.5%, the specificity 99.6%, and the positive predictive value 82%. The performance was similar across demographic subgroups.

**Conclusions:**

The WAI could be used to identify workers at high risk for prolonged sickness absence. However, due to low sensitivity many workers will be missed. Hence, additional factors are required to better identify workers at highest risk.

## Introduction

With an ageing population there is a need to retain healthy and productive workers. Prevention of, especially long-term, sickness absence can contribute to this goal. Since workers with multiple episodes or long-term sickness absence have an increased risk for mortality and are more likely to exit the labour force through disability benefits and unemployment [1–6], it would be helpful to identify high-risk workers before sickness absence occurs.

Several studies have suggested that the work ability index (WAI) could be used as a predictive tool to identify workers at high risk for long-term sickness absence or disability benefits [7–9]. The WAI is a frequently used tool in occupational health to assess a person’s work ability on a sum score between 7 and 49 in order to prevent temporary or permanent exit from work [10,11]. There is evidence that poor or moderate work ability, as compared to excellent work ability, is related to, especially long-term, sickness absence. Kujala et al (2006) reported an elevated risk for > 9 days of sickness absence among employed Finnish men and women with poor or moderate work ability in their early 30s (odds ratios ranging from 2.10 to 5.47) [8]. Alavinia et al (2009) showed similar findings among construction workers (rate ratios ranging from 2.35 for moderate spells of sick leave, to 3.76 for long spells) [12].

However, these associations do not provide insight into how well the WAI discriminates between workers with and without future sickness absence. Therefore, quantification of the discriminative ability of the WAI is needed. Among construction workers, the WAI discriminated adequately between workers at high-risk and low-risk for future disability benefits with an area under the curve (AUC) of 0.78 [13]. Among workers with chronic back pain, similar findings were reported. Those with a WAI score of 20 points or less had a 16-fold higher risk for disability benefits than those with a higher WAI score (AUC 0.80) [14]. At this moment, information about the prognostic value of the WAI to discriminate between workers with different durations of sickness absence is scarce. To the knowledge of the authors there is only one study which investigates whether the WAI is a suitable screening instrument for long-term sickness absence [15]. Furthermore, it remains unknown whether the discriminative ability differs across age, sex, and education groups, which are found to be important individual determinants of sickness absence [16]. This study aims to investigate 1) the prognostic value of the WAI in the prediction of sickness absence, and 2) whether the discriminative ability differs across sex, age, and educational groups.

## Methods

### Study design and population

This longitudinal study with 12-months follow-up is part of a larger study aiming to gain insight into the impact of a web-based health promotion programme on absenteeism [17]. Workers from a Dutch financial services company who completed participation in a Health Risk Assessment (HRA), called PreventionCompass [17], were included in this study. Pregnant women were excluded from participation in the HRA. Based on a previous reported participation level of 34% [18], it was estimated that approximately 11,000 workers were invited to participate in the HRA between August 1 2007 and July 1 2009. The sickness absence register was made available for the study period only. Therefore, to allow a full follow-up of 12 months, only workers who enrolled in the HRA before June 30 2008 were eligible for participation in the current study. Because of the limited capacity for onsite collection of biometric measurements, workers were gradually invited to participate in the HRA. A total of 3,826 workers participated in the HRA between August 1 2007 until July 1 2009. Of them, 1,351 participated before June 30 2008 and were eligible for participation in this study. Complete information on sickness absence, work ability, and individual characteristics was available for 1,331 workers.

At inclusion a web-based health questionnaire was completed (30–45 minutes), biometric measurements (height, weight, waist circumference, blood pressure) were taken, and laboratory samples were tested. The health questionnaire included questions on individual characteristics, work, lifestyle, personal health history, family risk, and psychological health factors. Based on the health questionnaire completion date, a 12-month follow-up period was defined for each participant in the study. Workers with incomplete data on sickness absence, work ability, or individual characteristics were excluded from the analyses.

### Ethical statement

Participants were invited by the company’s human resources department, who sent anonymous emails based on a random selection of workers by month of birth. The invitation e-mail included information on the HRA and informed workers that participation was voluntary and at no cost, that all personal information would be treated confidentially, and that no individual results would be shared with their employer or other parties [17]. A single reminder was sent after two weeks.

Observational research with questionnaire data does not fall within the ambit of the Dutch Act on research involving human subjects and does not require the approval of an ethics review board. In accordance with the requirements for identifiable data collection in the Dutch Code of Conduct for Observational Research digital informed consent was obtained from all study participants as part of the activation procedure of their online PreventionCompass account [19]. NIPED does collect and process personal information in the HRA. However, NIPED acts in accordance with the Dutch data protection act, in its use of personal details. Only anonymized data were made available to the researchers of the Erasmus MC.

### Sickness absence

During the study period, the sickness absence register was maintained by the occupational health service, which registered frequency and duration of every absence episode. Sickness absence was registered as calendar days from the first sick day onwards. In the analyses, absence episodes of three days or more were multiplied by the fraction of employment for part-time workers. The total number of sick days during the one year follow up period was categorised into 0 days (no absence), 0<days<5 (less than one week), 5≤days<15 (between 1 and 2 weeks), and ≥ 15 days (more than 2 weeks). These categories reflect different actions undertaken in the disease management, varying from a single contact by the supervisor to involvement of the occupational health physician. Specific diagnosis of the disease resulting in sickness absence was not made available to the researchers.

### Work ability

Work ability was measured using the WAI, which has been shown to be a valid, reliable, and cross-national instrument for use in occupational health [20–22]. The WAI consists of 7 dimensions. Dimension 1 asks to indicate on a scale from 0 (not able to work) to 10 (lifetime best) to estimate the current work ability compared with the lifetime best. Dimension 2 contains two questions and assesses on a 5-point scale ranging from 1 (very poor) to 5 (very good) the subjective current work ability in relation to the physical and mental demands of work (sum score of dimension 2 ranges from 2–10). Dimension 3 assesses the number of diseases diagnosed by a physician. To prevent overlap in the HRA questionnaire, the presence of diseases was ascertained using questions on the personal health history instead of the list of 14 diseases asked in the WAI (i.e. injury due to accident, musculoskeletal disease, cardiovascular disease, respiratory disease, mental disorder, neurological or sensory disease, digestive disease, genitourinary disease, skin disease, tumour or cancer, endocrine or metabolic disease, blood disease, heritable disease, other disorders/diseases). The questions on personal health history encompassed all disease categories included in the WAI, except skin disease and heritable diseases. The number of diseases are accumulated, and scores for this dimension range from 1 (≥5 diseases) to 7 (0 diseases). Dimension 4 assesses on a six point scale ranging from 1 (fully impaired) to 6 (no impairments) the subjective estimation of work impairments due to disease by asking whether the illness is a hindrance in their job. Dimension 5 concerns the number of days off work due to sick leave in the previous 12 months, with answering categories ranging from 0 days (5) to 100 days or more (1). Dimension 6 asks ‘Do you believe, according to your present state of health, that you will be able to do your current job two years from now?’. A score of 1 (hardly able to work), 4 (not sure), or 7 (fairly sure) could be obtained. Dimension 7 assesses the mental resources in the past few months using three questions concerning enjoying regular daily activities, being active and alert, and feeling to be full of hope about the future, with answering categories ranging from ‘never’ (0) to ‘always’ (4). For dimension 7 a sum score was calculated, leading to a score of 1 (if the sum score ranged between 0–3 points) to 4 (if the sum score ranged between 10–12 points) [23,24]. The total WAI score was calculated as the sum score of the 7 dimensions and ranges from 7–49. The WAI was categorised into “poor” (7–27), “moderate” (28–36), “good” (37–43), and “excellent” (44–49) work ability [24]. The WAI score was converted so that the highest value (49) represented the poorest work ability and the lowest value (7) the best work ability in order to describe that lower work ability is a risk factor for a greater number of sick days. The converted score was used in all analyses, except for the descriptive statistics, to facilitate the interpretability of the results.

### Individual factors

Information on sex, age, and education was collected in the baseline questionnaire. Age in years was categorised into three groups: < 40 years, 40–49 years, and ≥ 50 years. Education was measured as the highest educational level ever completed and classified into three groups: high (higher vocational schooling, or university), intermediate (higher secondary schooling, or intermediate vocational schooling), and low (primary school, lower and intermediate secondary schooling, or lower vocational schooling).

### Statistical analysis

For the main variables descriptive statistics were generated, i.e. numbers and percentages for dichotomous and categorical variables, and means and standard deviations for continuous variables. Univariate and multivariable multinomial logistic regression analyses were used to assess the association of the WAI score and individual characteristics with the occurrence of sickness absence. To assess the fit of the multinomial model a post estimation for goodness-of-fit was performed [25]. Although sickness absence is an ordinal variable, a multinomial regression analysis rather than an ordinal logistic regression analysis was performed to gain insight into the associations across the specific sickness absence categories. Previous studies have shown that short-term and long-term sickness absence are different types of sickness absence and have different determinants [6,16,26,27]

To assess the ability of the WAI to discriminate between workers with different durations of sickness absence, pairwise AUC’s were estimated. The pairwise AUC compares each pair of categories using only those workers that belong to one of the two categories at hand [28]. For our study there are four categories of sickness absence, hence, six pairwise AUC’s were estimated. To determine the overall ability of the WAI to discriminate correctly between these categories of sickness absence the ordinal c-index (ORC) was estimated as the average of all pairwise AUC’s [29]. The 95% confidence intervals (95%CI) were estimated with bootstrapping using 200 bootstrap replications [29]. The ORC is an attractive measure since it summarises the discriminative ability for ordinal outcomes into one single metric that can be compared directly with the AUC measure for a dichotomous outcome. The ORC is similarly interpreted as the traditional AUC: a value of 0.5 indicates a discriminative ability not better than chance, an AUC of 1.0 indicates perfect discrimination [29]. The discriminative ability was also estimated for each separate WAI dimension. Additionally, the discriminative ability of the WAI was assessed after removing the single dimensions in order to explore whether the prognostic value of the WAI was mainly driven by one of its dimensions. To assess whether the discriminative ability of the WAI was different across sex, age, and educational groups, subgroup analyses were performed.

Test characteristics (sensitivity, specificity, positive predictive value (PPV), and negative predictive value (NPV)) of the total WAI score were assessed at the cut-off between poor and moderate work ability (≤ 27) and at the cut-off between moderate and good work ability (≤ 36). ROC curves were created to calculate the AUC. Subsequently, sensitivity and specificity were assessed for different numbers of sick days (0 days vs. ≥ 1 days; < 5 days vs. ≥ 5 days; < 15 days vs. ≥ 15 days). The PPV was estimated as the true positives divided by the total positives. The NPV was estimated as the true negatives divided by the total negatives.

All analyses were conducted with SPSS 20.0 for Windows (IBM Software, Chicago), except for the calculation of the 95%CI around the ORC, which were calculated with R, version (R_3.0.3.tar.gz) (R Foundation for Statistical Computing, Vienna). Confidence intervals for the test characteristics were estimated using efficient-score method, described by Newcombe [30,31]. A p-value <0.05 was considered statistically significant.

### Results

The characteristics of the study population are presented in Table 1. Nearly half of the population was female (46.8%), and age ranged from 21–62 years, with a mean of 43.3 years (±8.9 years). One fifth of the population had a low educational level (20.4%).

**Table 1:** Characteristics of study sample consisting of office workers (n = 1,331).

		n (%) or mean ±sd
**Work ability and sickness absence**		
Work ability index (7–49)^a^		42.1 ±4.8
Work ability index category	Excellent	589 (44.3)
	Good	594 (44.6)
	Moderate	122 (9.2)
	Poor	26 (2.0)
Cumulative number of sick days during 1 year	0 days	266 (20.0)
	0<days<5	404 (30.4)
	5≤days<15	401 (30.1)
	≥ 15 days	260 (19.5)
**Individual factors**		
Sex, female		623 (46.8)
Age	< 40	490 (36.8)
	40–50	480 (36.1)
	≥ 50	361 (27.1)
Education	High	561 (42.1)
	Intermediate	499 (37.5)
	Low	271 (20.4)

n: number of workers; sd: standard deviation.

^a^ higher scores indicate better work ability.

### WAI as a determinant of sickness absence

During the follow-up period, 80% of the study population had at least one day of sickness absence. Almost one fifth (19.5%) was absent from work ≥ 15 days due to sickness (Table 1). Table 2 shows that workers with lower work ability were more likely to have sickness absence (OR per point lower WAI score: 1.10, 1.13, and 1.27 for 0<days< 5, 5≤days<15, and ≥ 15 days versus 0 days). The goodness-of-fit analysis of the multivariable multinomial regression analysis showed that the estimated probabilities and observed probabilities did not significantly differ (p-value = 0.11), and thus was sufficient.

**Table 2:** Univariate and multivariable multinomial regression analyses with odds ratios and 95% confidence intervals for the association between work ability, and individual factors with sickness absence among office workers (n = 1,331).

		Univariate			Multivariable		
		Sickness absence			Sickness absence		
		0<days<5^a^	5≤days<15^a^	≥ 15 days^a^	0<days<5^a^	5≤days<15^a^	≥ 15 days^a^
		OR (95% CI)	OR (95% CI)	OR (95% CI)	OR (95% CI)	OR (95% CI)	OR (95% CI)
**Work ability index**							
Work ability index score (7–49)^b^		1.10* (1.05–1.15)	1.13* (1.08–1.18)	1.27* (1.21–1.33)	1.11* (1.06–1.16)	1.15* (1.10–1.20)	1.27* (1.21–1.33)
**Individual factors**							
Sex, female		1.56* (1.14–2.13)	1.39* (1.01–1.90)	1.51* (1.07–2.14)	1.23 (0.87–1.73)	1.06 (0.75–1.50)	1.15 (0.77–1.70)
Age	< 40 years	1	1	1	1	1	1
	40–50 years	0.75 (0.52–1.08)	0.82 (0.57–1.19)	1.30 (0.85–1.98)	0.71 (0.48–1.05)	0.75 (0.51–1.11)	0.99 (0.62–1.57)
	≥ 50 years	0.49* (0.33–0.73)	0.53* (0.36–0.78)	1.16 (0.75–1.78)	0.42* (0.27–0.65)	0.41* (0.26–0.64)	0.62 (0.37–1.05)
Education	High	1	1	1	1	1	1
	Intermediate	0.98 (0.69–1.38)	1.14 (0.81–1.61)	1.91* (1.29–2.84)	0.94 (0.65–1.34)	1.07 (0.75–1.54)	1.53 (1.00–2.35)
	Low	1.17 (0.76–1.79)	1.16 (0.75–1.79)	2.78* (1.74–4.43)	1.29 (0.80–2.08)	1.24 (0.77–2.02)	2.10* (1.23–3.58)

*p-value <0.05; OR: odds ratio; 95% CI: 95% confidence interval; n: number of workers.

^a^0 days of sickness absence is reference category.

^b^lower scores indicate better work ability.

When using the traditional four categories of the WAI index, there was a clear upward trend for lower WAI categories with larger odds ratios for greater number of sick days, i.e. workers with a poor/moderate (OR 15.14, 95% CI 7.69–29.81), or good (OR 4.12, 95%CI 2.77–6.14) work ability had a higher likelihood on ≥ 15 sick days than workers with excellent work ability (S1 Table).

### Prognostic value of the WAI

**Fig 1:**
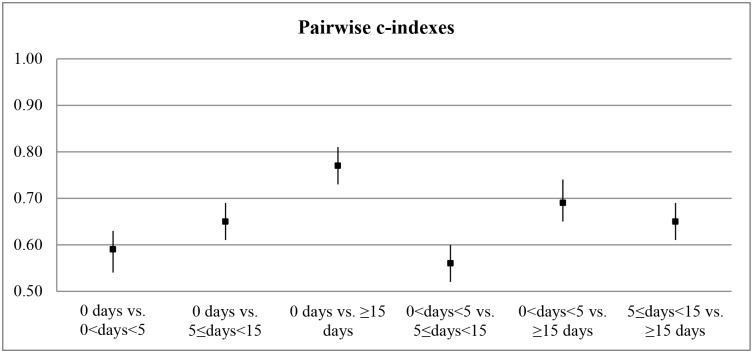
Pairwise AUCs: ability of the work ability index (WAI) to discriminate between categories of sickness absence. AUC: Area under the curve.

The ORC was 0.65 (95%CI 0.63–0.68), representing a 65% probability that the WAI correctly separates two cases from two randomly chosen categories of sickness absence. Fig 1 shows that the WAI fails to separate workers without sickness absence from workers with 0<days<5 of absence. The WAI could best discriminate between workers with 0 or 0<days<5 sick days and workers with ≥ 15 sick days (AUC 0.77, and 0.69 respectively).

Dimension five (i.e. sickness absence in previous year) had the best discriminative ability (ORC 0.67, 95%CI 0.65–0.70) as compared to the other WAI dimensions (Table 3). Excluding dimension five from the total WAI score resulted in a minor decrease in ORC from 0.65 to 0.63 (Table 3). Exclusion of other dimensions also resulted in minor changes in ORC of 0.01, or no change at all.

**Table 3:** Discriminative ability of the WAI dimensions in the prediction of different durations of sickness absence among office workers (n = 1,331).

	Single dimensions^a^	Exclusion analyses^b^
	ORC (95% CI)	ORC (95%CI)
Dim 1. Subjective work ability	0.59* (0.55–0.61)	0.66* (0.64–0.68)
Dim 2. Work ability in relation to demands	0.60* (0.57–0.62)	0.65* (0.63–0.67)
Dim 3. Number of diseases	0.59* (0.56–0.61)	0.65* (0.62–0.67)
Dim 4. Work impairments	0.58* (0.56–0.60)	0.65* (0.63–0.67)
Dim 5. Sick leave past year	0.67* (0.65–0.70)	0.63* (0.60–0.65)
Dim 6. Prognosis of work ability	0.52* (0.51–0.54)	0.65* (0.63–0.68)
Dim 7. Mental resources	0.56* (0.54–0.58)	0.65* (0.63–0.68)

*p-value <0.05; ORC: ordinal c-index; 95% CI: 95% confidence interval; Dim: dimension.

^a^discriminative ability of the single WAI dimensions.

^b^discriminative ability of the total WAI score minus a dimension.

**Fig 2:**
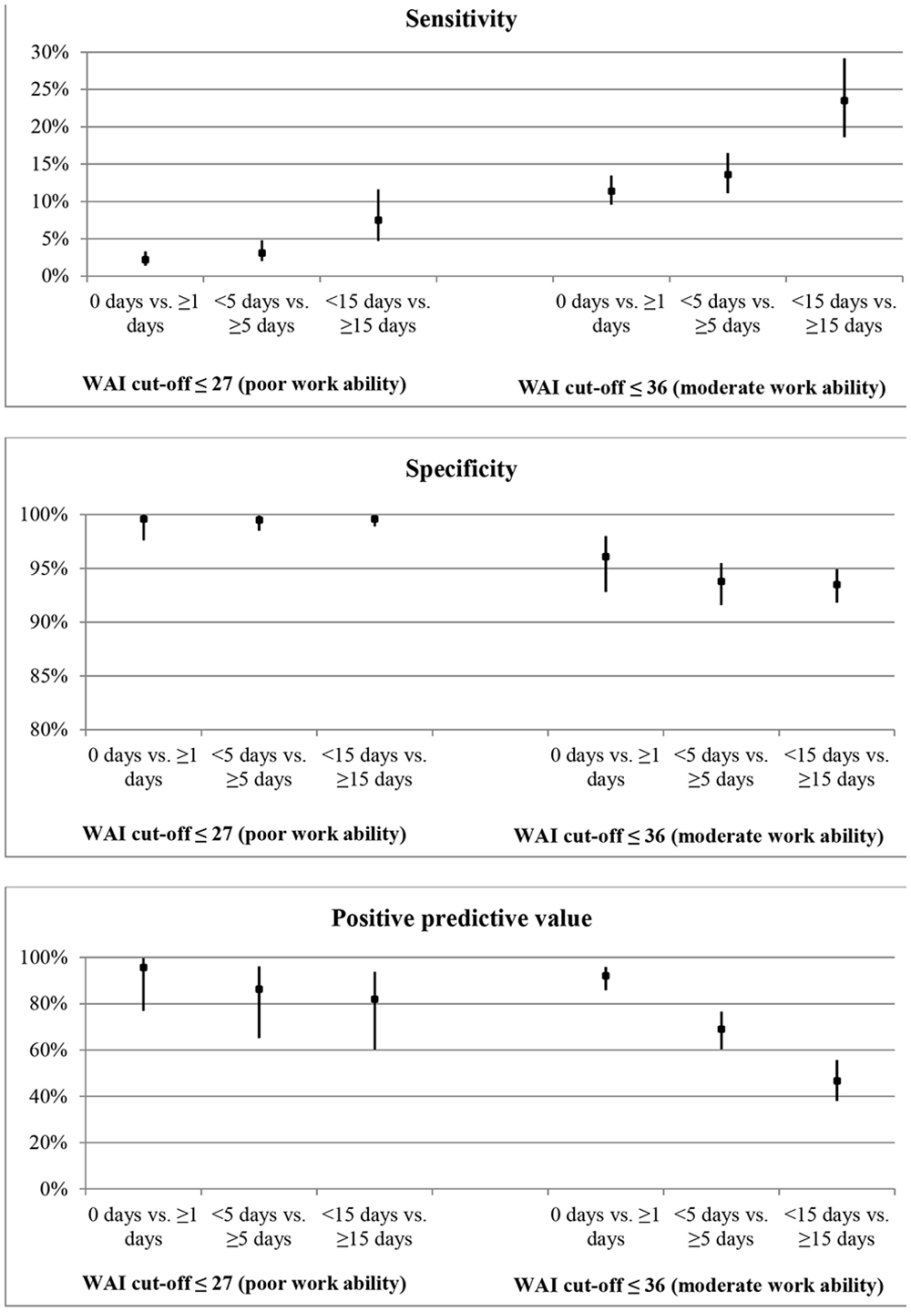
Test characteristics (sensitivity, specificity, and positive predictive value) at different cut-off values of the work ability index (WAI) for different durations of sickness absence.

Fig 2 presents the test characteristics of the WAI for predicting different numbers of sick days. At the cut-off between poor and moderate work ability (score ≤ 27) the sensitivity was 7.5%, specificity 99.6%, and PPV 82.0% (NPV was also 82.0%) for < 15 sick days vs. ≥ 15 sick days. At the cut-off between moderate and good work ability (score ≤ 36) sensitivity increased to 23.5%, but specificity and PPV decreased to 93.5% and 46.7%, respectively, for <15 sick days vs. ≥ 15 sick days.

### Discriminative ability of the WAI for sickness absence across subgroups

The ability of the WAI to discriminate between different categories of sickness absence was similar for male and female office workers (ORC 0.66 versus 0.64), as well as for different age groups (ORC 0.65). No differences in discriminative ability were observed between educational groups (high: ORC 0.66, 95%CI 0.61–0.71, intermediate: ORC 0.62, 95%CI 0.59–0.66, and low: ORC 0.66, 95%CI 0.62–0.71) (S2 Table).

## Discussion

Until now, information about the prognostic value of the WAI was scarce. In this study the WAI showed a reasonable ability to discriminate between different durations of sickness absence. The WAI could fairly well identify workers with prolonged sickness absence from workers without absence or with low absence. This confirms that the WAI could be used to identify workers at risk for prolonged sickness absence. Although the WAI had a high specificity, its sensitivity was low. Due to this low sensitivity, not all workers with prolonged sickness absence are identified by a low work ability score. The discriminative ability did not differ between demographic subgroups.

In the Netherlands, workers are required to call in sick for work to their employer on the first day of their absence. A worker will be paid at least 70% of his/her full salary during the first two years of sickness absence. A worker is eligible for disability benefit after the first two years of sickness absence [8,32,33]. In our study population 19.5% of the participants had prolonged sickness absence (≥15 days) during the follow-up year. This is comparable with the findings of other Dutch studies (16–21%) [12,26,34]. However, 80% of our participants had at least one day of sickness absence. Other Dutch studies reported lower prevalences of sickness absence (34–58%) [12,26,34]. These differences in short-term sickness absence may be explained by differences in study population and measurement method.

Previous studies found that lower work ability was associated with sickness absence [8,12,35], disability benefits [22], and productivity loss at work [36]. This study confirmed that a lower WAI score was related to sickness absence, especially ≥ 15 sick days. Moreover, we found that the WAI was able to correctly discriminate between four categories of sickness absence in 65% of the cases. Discrimination between workers without sickness absence and workers with ≥ 15 sick days was even better (77% correct classification of the cases). A recent study found a very similar ability of the WAI to discriminate between workers with more than 14 sick days and those with less than 14 sick days (AUC 0.78)[15]. Besides, this trend in discriminative ability is similar to the earlier reported trend in associations between a lower work ability score and sickness absence, which are systematically stronger for a greater number of sick days [8,12,35].

The results of this study show that the overall ability of the WAI to discriminate between the four categories of sickness absence was reasonable. The WAI discriminated best between 0 or 0<days<5 sick days versus ≥ 15 sick days. This indicates that the WAI could be used as a tool to identify office workers at future risk for prolonged sickness absence. However, at the cut-off between poor and moderate work ability only 7.5% of the workers with ≥ 15 sick days were identified by their poor work ability score. On the other hand, the PPV of 82% indicates that from all workers with a poor work ability score at baseline, 82% had ≥ 15 sick days at follow-up. For the practical use of the WAI in public and occupational health care this suggests that workers with a poor work ability score are highly likely to have ≥ 15 sick days in the next 12 months, but that many workers with sickness absence of two weeks or more will not be identified by a poor work ability score. Hence, additional factors are required to better identify workers at highest risk for prolonged sickness absence. Introducing a higher cut-off in the WAI, for example between moderate and good work ability, might also be a solution. It improves the sensitivity to 24%, however, at the expense of lower specificity and PPV.

Since workers with poor work ability are highly likely to have prolonged sickness absence in the next 12 months, interventions aiming at prevention of prolonged sickness absence could be targeted at workers with poor (and moderate) work ability. However, one has to recognize that many workers at risk for prolonged sickness absence will be missed when only focusing on the WAI score. Therefore, additional information on risk factors for sickness absence, such as on the private situation, organizational factors, or on physical and psychosocial work related factors, and lifestyle related factors, may be needed to better identify those workers at highest risk.

To the knowledge of the authors this is the first study that evaluated the prognostic value of the individual dimensions of the WAI. The first dimension of the WAI (i.e. self-assessed current work ability, range 0–10) has often replaced the WAI in clinical and population-based studies [37,38]. A Swedish study concluded that the first dimension of the index and the full WAI had a very strong correlation (Spearman r = 0.87) and showed similar associations with degree of sick leave [39]. However, our study indicates that the whole index has a somewhat better discriminative ability (ORC 0.65) than the first dimension of the WAI (ORC 0.59). Among construction workers, Roelen et al (2014) found similar results, whereby the first dimension had a fair discriminative ability (AUC = 0.67), while the total index had an adequate discriminative ability (AUC = 0.78) to identify workers at risk for disability benefit [13]. The differences in outcomes between the studies may be explained by the fact that the Swedish study focused on correlations and associations while our study and that of Roelen et al (2014) focused on the discriminative ability. Furthermore, the Swedish study consisted of female workers who were already on long-term sick leave, while our study consisted of male and female office workers. This may also have contributed to different findings.

Despite the fact that a previous review reported an association between individual characteristics and sickness absence [16], there were no large differences in the discriminative ability of the WAI across age, sex, and educational groups. This indicates a generic discriminative ability of the WAI in the identification of sickness absence.

### Strengths and limitations

Some strengths and limitations need to be considered. The large study population, longitudinal data, and register-based information regarding the number of sick days are strengths of this study [40,41]. A first important consideration is the fact that the WAI includes information on previous sickness absence. From other studies it is known that sickness absence in previous years is a predictor for future sickness absence [42,43]. However, the exclusion analysis indicated that the discriminative ability of the WAI was not fully driven by this single dimension. Second, selection bias cannot be ruled out, since it is not clear whether the respondents are a representative sample of all workers in the financial service company. Overall, participation in the HRA was not selective for education, gender, and age [44]. Therefore, the potential effect of this bias is considered low. Third, this study was conducted among office workers (i.e. white-collar workers) in the financial industry in midst of the global financial crisis. Therefore, it remains unknown whether our results are generalizable to occupations with a higher physical workload (i.e. blue-collar workers). However, it is likely that results will be comparable since studies on the association between work ability and sickness absence show similar results for both occupational groups [8,12]. Fourth, the presence of diseases diagnosed by a physician was ascertained using the questions on personal health history rather than the original third dimension of the WAI. The items on personal health history encompassed all disease categories included in the original WAI index question, except for skin disease and heritable diseases. This may have resulted in a slightly lower disease prevalence and hence a slightly higher WAI score. Last, when interpreting the results one has to keep in mind that this tool is aimed at a selective primary prevention strategy and should be used only within workplaces where workers have sufficient employment protection against health-related redundancy policies.

## Conclusion

Until now, information about the prognostic value of the WAI was scarce. From this study we can conclude that the work ability index is able to identify workers with prolonged sickness absence fairly well. This indicates that the WAI could be used to identify workers at high risk for prolonged sickness absence. However, due to a low sensitivity, not all workers with prolonged sickness absence are identified by a low work ability score. Hence, additional factors are required to better identify workers at risk for prolonged sickness absence.

## Supporting Information

S1 Table Univariate multinomial regression analyses with odds ratios and 95% confidence intervals for the association between categories of work ability with sickness absence among office workers (n = 1,331). *p-value <0.05; OR: odds ratio; 95% CI: 95% confidence interval; n: number of workers.^a^0 sick days is reference category.
(PDF)

S2 Table Discriminative ability of the WAI in the prediction of different durations of sickness absence among office workers, subgroup analysis (n = 1,331). *p-value <0.05; WAI: Work ability index; ORC: ordinal c-index; 95% CI: 95% confidence interval.
(PDF)
