# Supplementary material for: The Prognostic Value of the Work Ability Index for Sickness Absence among Office Workers
Source: PLoS One. 2015 May 27;10(5):e0126969. doi: 10.1371/journal.pone.0126969 (PMC4446207; doi:10.1371/journal.pone.0126969)
Supplement: S1 Table — *p-value <0.05; OR: odds ratio; 95% CI: 95% confidence interval; n: number of workers.a0 sick days is reference category. (PDF) [file pone.0126969.s001.pdf]

	Sickness absence		
	**0<days<5[a]**	**5≤days<15[a]**	**≥ 15 days[a]**
	OR (95% CI)	OR (95% CI)	OR (95% CI)
**Work ability categories**			
Excellent	1	1	1
Good	1.80* (1.29-2.51)	2.77* (1.99-3.87)	4.12* (2.77-6.14)
Poor/moderate	2.38* (1.19-4.74)	3.20* (1.60-6.38)	15.14* (7.69-29.81)
