# Supplementary material for: The Prognostic Value of the Work Ability Index for Sickness Absence among Office Workers
Source: PLoS One. 2015 May 27;10(5):e0126969. doi: 10.1371/journal.pone.0126969 (PMC4446207; doi:10.1371/journal.pone.0126969)
Supplement: S2 Table — *p-value <0.05; WAI: Work ability index; ORC: ordinal c-index; 95% CI: 95% confidence interval. (PDF) [file pone.0126969.s002.pdf]

		Discriminative ability
		ORC (95% CI)
Sex	Male	0.66* (0.61-0.69)
	Female	0.64* (0.61-0.68)
Age	< 40	0.65* (0.60-0.69)
	40-50	0.65* (0.62-0.69)
	≥ 50	0.65* (0.61-0.70)
Education	High	0.66* (0.61-0.71)
	Intermediate	0.62* (0.59-0.66)
	Low	0.66* (0.62-0.71)
